# Understanding barriers to breast screening: an online survey of non-attenders as part of a service evaluation in the breast screening programme in England

**DOI:** 10.1186/s12889-025-23691-3

**Published:** 2025-07-19

**Authors:** Gemma Hutton, Shuping J. Li, Samantha L. Quaife, Adam Brentnall, Jacqui Cookson, Jacquie Jenkins, Sue Hudson, Sharon Webb, Emma O’Sullivan, Stephen W. Duffy, Judith Offman, Jo Waller

**Affiliations:** 1 Wolfson Institute of Population Health, Queen Mary University of London, London, UK; 2 Primary Care, Community, Vaccinations & Screening Directorate, NHS England, London, UK; 3 Peel & Schriek Consulting Ltd, London, UK

**Keywords:** Breast Screening, Mammogram, Barriers, Survey, Uptake, Acceptability, Breast cancer, Attendance

## Abstract

**Background:**

Early detection of breast cancer through organised mammography screening of asymptomatic individuals reduces breast cancer mortality. Breast screening is offered every three years to women aged 50 to 71 years in England. However, over a third of eligible women did not attend in 2022–2023. Understanding reasons for non-attendance is critical to ensuring the effectiveness of the breast screening programme by highlighting ways to improve access to screening.

**Methods:**

As part of a service evaluation in the NHS Breast Screening Programme (NHSBSP), we conducted a cross-sectional survey using an online questionnaire in February 2024. Participants were non-attenders from 15 NHSBSP services in England. All women invited to breast screening who subsequently did not attend in October or November 2023 and had a valid mobile number recorded on their records received a single text message containing a link to the survey. The online survey gathered demographic and basic screening history information and assessed endorsement of common barriers to breast screening. Descriptive analysis was used to identify the most commonly endorsed barriers and chi-squared tests were used to explore demographic variation in barrier endorsement.

**Results:**

Overall, 27,729 women were identified as not attending a screening appointment in October or November 2023. Of these, 17,221 had mobile numbers and were sent a text message inviting them to participate in the survey. In total, 1,074/17,221 (6%) participants completed the survey. The most frequently endorsed barriers to breast screening were: difficulties making a convenient appointment (30%), concern that a man may do the mammogram (28%), worry about the mammogram being painful (27%), previously experiencing pain during a mammogram (26%), having too many other things to worry about (25%) and the appointment being located too far away (23%). Endorsement of the most common barriers varied significantly by age, ethnicity, mental health status and disability but not by educational level.

**Conclusions:**

These findings identify barriers that could be targeted to increase screening uptake including increasing appointment availability and proximity, reinforcing the message that breast screening is a female-only environment as well as developing interventions to reduce and manage pain during breast screening.

**Supplementary Information:**

The online version contains supplementary material available at 10.1186/s12889-025-23691-3.

## Background

Early detection of breast cancer through organised mammography screening of asymptomatic individuals reduces breast cancer mortality [1, 2]. In the UK, the NHS Breast Screening Programme is a national programme offering routine breast screening every three years to women aged 50–71. After the age of 71, women can still be screened by self-referring to their local screening service. Women who are currently undergoing breast cancer treatment or who are less than 5 years post-treatment for breast cancer are screened separately and are not invited to routine screening. Women typically receive postal invitations from their local breast screening service which are first sent to women aged between 49 and 8 months and 53, and automatically continue 3-yearly until age 71. These invitations may either specify a date, location and time for the appointment which can be rescheduled by the recipient if required or may invite the recipient to contact the service to schedule an appointment. The invitations are accompanied by a leaflet providing a short description of the screening process [3]. At least one text-message reminder is sent before an appointment date. Women who do not attend a scheduled appointment are sent a second invitation letter within 2–3 weeks of the original appointment. Text message reminders are sent to women who do not attend. The timing of each reminder is determined by the local breast screening service.

An effective screening programme relies upon high uptake by the target population. The most recent NHS screening statistics reported that 35% of eligible women in England did not attend breast screening in the year 2022–2023 [4]. Although some women may make an informed choice not to take part, there are many other potentially modifiable reasons for non-participation. Better understanding of these reasons could help to inform continued improvements to service delivery and provide a basis for targeted interventions to increase uptake thereby increasing the effectiveness of the breast screening programme.

The decision to attend breast screening is multi-faceted and research has identified numerous barriers that may reduce screening uptake. We define barriers as *“factors that hinder*,* limit*,* or prevent people from engaging in a certain behaviour”* [5], which can include individual and environmental influences on behaviour, in line with the Integrated Screening Action Model [6]. Uptake in England varies according to age, region and type of invitation with higher uptake identified in older women aged > 65 years, in the Southeast and in women receiving a routine invitation to screening where they have attended a previous screen within the last 5 years [4]. Uptake is also lower in areas of higher deprivation, and in women from minoritised ethnic backgrounds and those who have a disability [7–11]. Research has also identified several modifiable barriers to screening attendance including logistical, emotional and attitudinal barriers. A population-based survey including 945 women eligible for breast screening, carried out by Cancer Research UK in September 2023, identified that the most commonly reported barriers to screening related to difficulties making and attending an appointment, concerns about breast screening being painful, beliefs that the harms of screening outweigh the benefits as well as misperceptions about the mammographer being male and screening only being beneficial for symptomatic women [12]. However, population-based surveys typically under-represent those who are overdue for screening and the voices of non-attenders are seldom heard within research. The sample used in the Cancer Research UK survey predominantly consisted of screening attenders with only a small number of non-attenders (*N* = 91). Prior to conducting this evaluation there were no large-scale surveys of breast screening non-attenders in England and there was a need to ascertain an up-to-date overview of the barriers that may prevent attendance.

As part of a service evaluation commissioned by NHS England, a national online survey of non-attending women was conducted and aimed to provide current insight into the barriers preventing women from attending breast screening to highlight key areas that could be targeted to increase screening uptake.

## Methods

### Aim

Our aim was to determine the barriers to attending breast screening reported by women who had not attended following a recent screening invitation and to identify any demographic variation in barrier endorsement.

### Design

As part of a service evaluation, a link to a cross-sectional online survey of barriers to breast screening was sent to non-attenders via text message. Eligible non-attending women received a single text message from their local Breast Screening Service which contained a hyperlink to the survey. The text message contained the following information:

- *Breast screening uptake is falling. We want to know why some women don’t come to breast screening so we can find ways to help more women attend. Please could you fill in a short survey via the link below. It should only take 5 min and all answers are anonymous.*

The text messages were sent over a two-week period between 1st and 14th February 2024.

### Setting

All eligible breast screening services in England were invited to participate in the service evaluation by NHS England. Eligibility for participation included having more than 100 non-attending women per month and having sufficient text messaging capability to be able to send a single text message to non-attending women during the recruitment period. A total of fifteen breast screening services took part in the study (see Additional file 1 for a full list). Participating services were well distributed across England and included a range of urban and rural areas.

### Participants

Women invited to routine breast screening (i.e. registered with a GP, aged between 49 and 8 months and 70 years and 364 days, without a breast cancer diagnosis in the last 5 years) with a valid mobile telephone number recorded on NBSS (National Breast Screening System) who were recorded as not making or attending a breast screening appointment in October or November 2023, after receiving an invitation in the preceding 4–6 weeks, were sent a link to the survey in a single text message. This included women who had received a single invitation and those who had received an invitation and a reminder during this period.

Demographic and screening characteristic data from the invited population were extracted from NBSS. These characteristics were age, screening status ((prevalent (i.e. had never participated in breast screening, which included first-time invitees and previous non-attenders) or incident (women with at least one previous screen recorded)) and Index of Multiple Deprivation (IMD) quintile based on residential postcode. As the survey was fully anonymous, survey responses could not and were not linked with the data extracted from NBSS. The extracted data was used only to provide insight into the characteristics of the invited population.

### Survey measure

We developed an online survey (see Additional File 2 for the full survey) to assess barriers to screening attendance. Items were drawn from the literature, including the Cancer Awareness Measure [12]. Twenty barriers to screening were assessed using a binary fixed choice yes/no response option and included lack of awareness of breast screening, appointment inconvenience and logistical issues, pain, embarrassment, fear, concern that the harms of screening outweigh the benefits, poor health status, low risk perception, low trust, lack of perceived screening efficacy, low invitation acceptability and concern about COVID-19. A free text question was also included to enable women to provide any additional information or barriers.

Basic demographic variables including age-group, ethnic background, highest educational qualification, region, disability and mental health status were also collected. We included questions assessing basic screening information: awareness of breast screening, receipt of an invitation and prior attendance. For participants who indicated they were either unaware of breast screening, had never received an invitation to breast screening or who had recently attended a screening appointment, we collected data on demographic characteristics only, as barriers were not relevant.

Prior to circulation, all survey questions were evaluated by four female patient and public involvement (PPI) participants aged between 50 and 71. Each participant completed a think-aloud interview with a researcher to provide detailed feedback on the clarity, content and comprehensibility of the survey questions to ensure that questions were being understood and interpreted as planned. Minor changes to question wording were made based on this feedback. A pilot test with 20 further female PPI participants aged 50 to 71 was carried out to check timings and ensure acceptability, ease of use and functionality of the online survey.

The final survey was programmed in Microsoft Forms and hosted by NHS England. Fully anonymous data were transferred to Queen Mary University of London for analysis, under a data sharing agreement.

### Statistical analysis

Primary analysis measured the level of endorsement for each barrier, with 95% confidence intervals (CI). Exploratory analysis used χ^2^ tests to examine demographic variation in the six most endorsed barriers (defined as endorsement by > 20% of respondents), with a p value of < 0.05 reported as “significant” (no adjustment for multiple comparisons). Demographic analysis considered age, ethnicity, educational level, disability and mental health status because research has illustrated that these factors are associated with screening uptake [4, 7, 13–15]. Two demographic factor groups were collapsed for analysis due to a small number of participants within individual categories: white vs. all other ethnic groups, and degree level education or above vs. education below degree. Age was grouped into 5-year age bands: <55/55–59/60–64/65–69/70+.

For the free-text responses, we developed a coding frame based on both the identification of barriers that were already included in the survey and inductive codes that identified additional barriers. The coding frame was applied to all the free text responses by the first author. A proportion (59.0%) of the responses were also double coded by another independent researcher. Any discordant coding was discussed and resolved. Responses that contained existing barriers were aggregated into the main barrier responses in the survey. Additional barriers were described separately.

A priori, we aimed for a sample size of 1,400 completed surveys because it would give +/−2% precision (95% CI width) for barriers with 20% endorsement, which was considered sufficiently informative. Assuming approximately 60% of non-attenders would have mobile phone numbers available (information from NHS England) and a ~ 10% response rate for the survey, we planned to identify ~ 25,000 non-attenders to send invitations to ~ 15,000.

Data management and descriptive analysis were carried out using SPSS v29.0.

## Results

### Characteristics of the sample

A total of 27,729 women from the fifteen participating services were identified as not having attended breast screening in October or November 2023 after receiving an invitation in the preceding 4–6 weeks. A total of 10,508 women (37.9%) were excluded because they did not have a mobile phone number listed on NBSS. The remaining 17,221 women were sent the survey via text message. Therefore, we met our pre-planned sample size for invitations.

**Fig. 1:**
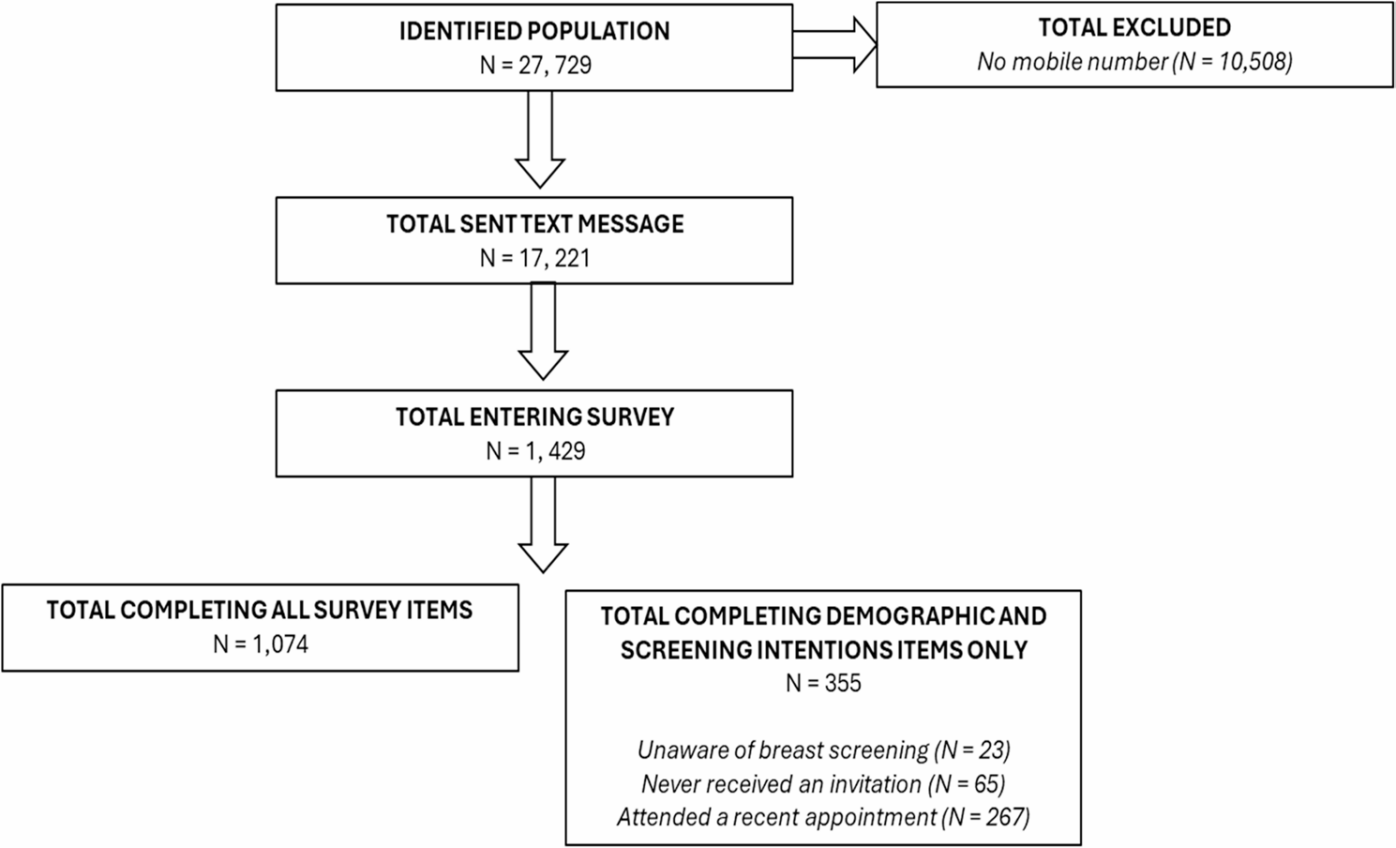
Progression of participants through the survey

Overall, 1,429 women entered the survey indicating a response rate of 5.1% of the total identified population and 8.3% of those sent a text message. A total of 1,074 women who indicated they were aware of breast screening, had received an invitation and had not attended an appointment recently completed all the survey items. A flow diagram showing the progression of women through the survey is shown in in Fig. 1.

### Demographic characteristics

#### Eligible population of non-attending women

A complete summary of the identified eligible population is reported in Additional file 3. Most non-attending women (21,531/27,729; 77.6%) were aged between 50 and 64, almost 60% (16,341/27,729; 58.9%) were from the two most deprived quintiles of deprivation and nearly two thirds (17,252/27,729; 62.2%) were prevalent screenees.

### *Survey respondents*

Most respondents reported being aware of screening (*N* = 1403/1429; 98.4%) and receiving a recent invitation (*N* = 1332/1429; 95.3%). A fifth reported attending a recent screening appointment (*N* = 267/1429; 19.9%). Examination of the demographic composition of those starting the survey (*N* = 1429) and those completing the full survey (*N* = 1074) revealed no major differences in any demographic characteristic. However, significantly fewer respondents from minoritised ethnic groups completed the barriers items compared with the number starting the survey. There was a 51.0% (75/147) loss of respondents from minoritised ethnic groups compared to a 22.0% (275/1248) loss of white respondents (see Additional File 4). Half of the respondents from minoritised ethnic groups were routed out because they reported being unaware of screening or reported not having been invited.

**Table 1:** Demographic characteristics of survey completers (*n* = 1,074)

	**N**	**%**
**Age (years)**		
< 55	303	28.2
55–59	314	29.2
60–64	261	24.3
65–69	148	13.8
70 +	41	3.8
Missing	7	0.7
**Ethnicity**		
White	973	90.6
Other ethnic groups (all)	72	6.7
* Mixed/Multiple*	15	1.4
* Asian/Asian British*	34	3.2
* Black/African/Caribbean/Black British*	16	1.5
* Other ethnic group*	7	0.7
Missing	29	2.7
**Highest Educational Qualification**		
Degree or above	341	31.8
Education below degree (all)	589	54.8
* NVQ or equivalent*	145	13.5
* AS*,* A-Levels or equivalent*	85	7.9
* GCSE. O-Levels or equivalent*	195	18.2
* Apprenticeship*	7	0.7
* Other qualifications*	76	7.1
* No qualifications*	81	7.5
Missing	144	13.4
**Disability**		
Yes, reported a disability	282	26.3
No disability	724	67.4
Missing	68	6.3
**Mental Health Condition**		
Yes, reported a mental health condition	242	22.5
No mental health condition	775	72.2
Missing	57	5.3
**English as first language**		
Yes	1013	94.3
No	61	5.7
**Region**		
North of England	263	24.5
Yorkshire and the Humber	288	26.8
Midlands	129	12.0
East/South East England (including London)	211	19.6
South West England	130	12.1
Missing	53	4.9
**Previously attended breast screening?**		
Yes (incident)	682	63.5
No (prevalent)	389	36.2
Missing	3	0.3
**Number of barriers endorsed (Maximum = 20)**		
0	139	13.0
1	186	17.3
2	196	18.2
3–6	433	40.3
7 or more	120	11.2

Demographic characteristics of respondents completing the survey are shown in Table 1. In summary, the majority were aged between 50 and 64 years (81.7%), with just under a third educated to degree level or above (31.8%). The women were predominantly white (90.6%) and English-speaking (94.3%), with just over a quarter reporting a disability (26.3%) and just under a quarter reporting mental health problem (22.5%). Almost two thirds of respondents reported previously attending attended breast screening (63.5%). Just over half were located in the North of England (51.3%).

### Endorsement of barriers to breast screening

Most respondents reported two or more barriers (69.7%) with 17.3% reporting a single barrier. A small proportion (11.2%) reported seven or more barriers (Table 1). The number and percentage of respondents endorsing each pre-specified barrier and mentioning the most common barriers identified in the free text responses are given in in Table 2, where the top six most-endorsed barriers were: (1) difficulty getting a convenient appointment (29.9%, 95% CI 27.2 to 32.7%) (2) concern about a man doing the screening test (28.3%, 95% CI 25.6 to 31.1%) (3) worry that breast screening would be painful (27.2%, 95% CI 24.6 to 30.0%) (4) previous experience of pain (25.9%, 95% CI 23.3 to 28.6%) (5) having more important things to worry about (23.8%, 95% CI 21.3 to 26.5%) and (6) the appointment being too far from home (22.7%, 95% CI 20.2 to 25.3%).

**Table 2:** Endorsement of barriers to breast screening (*N*; %; 95% confidence intervals) of all barriers (*N* = 1,074)

Barrier item	*N* (yes)	%	95% CI (%)
Logistical issues			
I found it difficult to get an appointment at a convenient time	321	29.9	27.2–32.7
I had other more important things to worry about than breast screening	256	23.8	21.3–26.5
The appointment was too far from home	244	22.7	20.2–25.3
I could not afford to cover the costs related to having an appointment	151	14.1	12.0–16.3
Embarrassment			
I didn’t want a man to do the screening test	304	28.3	25.6–31.1
I was worried about having to take my clothes off or that too much skin would be showing	166	15.5	13.3–17.8
I was too embarrassed to go to breast screening	161	15.0	12.9–17.3
Pain			
I was worried that breast screening might be painful	292	27.2	24.6–30.0
I have found breast screening painful or uncomfortable when I have been before	278	25.9	23.3–28.6
Fear			
I was too frightened of what the test might find	183	17.0	14.8–19.4
Health status			
I have a physical health condition or disability that makes it difficult for me to go to breast screening	181	16.9	14.7–19.2
I have a mental health condition or a learning disability that makes it difficult for me to go to breast screening	116	10.8	9.0–12.8
Low risk perception			
I had no symptoms of breast cancer	177	16.5	14.3–18.8
I don’t think I’m at risk of breast cancer	132	12.3	10.4–14.4
Harms vs. benefits			
Harms outweigh the benefits	165	15.4	13.3–17.7
Trust			
I don’t trust the NHS	88	8.2	6.6–10.0
I don’t think breast screening works	74	6.9	5.5–8.6
COVID-19			
I had symptoms that might have been related to COVID-19	69	6.4	5.0–8.1
I was worried about catching COVID-19 if I went for screening	57	5.3	4.0–6.8
Language			
Difficulty reading and understanding leaflet	14	1.3	0.7–2.2
Barriers coded from free-text responses			
Work commitments	91	8.5	6.8–10.3
Difficulties with appointment communication	78	7.3	5.8–9.0
Previous negative screening experience (not primarily related to pain or embarrassment)	55	5.1	3.9–6.6

We found a significant overlap in women reporting worry about pain and previous experience of pain. Among women who reported being worried about breast screening being painful (*N* = 292), just over half also reported a previous painful experience (*N* = 155, 53.1%). For women who did not report a previous painful experience (*N* = 796), only 17.3% (*N* = 137) reported being worried about pain. This suggests that previous painful experience is a particularly potent barrier as it may also increase the likelihood of worry about pain. It is notable that, in total, almost 40% (*N* = 415/1074) of respondents endorsed one of these pain-related barriers.

The top three most commonly described additional barriers identified within the free text responses were: (1) work commitments (8.5%, 95% CI 6.8 to 10.3%) (2), difficulties with appointment communication (7.3%, 95% CI 5.8 to 9.0%) and (3) a previous negative experience of screening (not primarily concerned with pain or embarrassment) (5.1%, 95% CI 3.9 to 6.6%).

### Analysis of free text responses

A free text response was entered by 842/1074 (78.3%) of respondents, providing additional insight into barriers to screening. Of these responses, 610/842 (72.4%) related to one of the pre-defined barrier items and were aggregated into the total barrier endorsement figures. Pain was the most common pre-defined barrier referred to within the free text responses accounting for 96/610 (15.7%) of these free text responses. Comments about pain related to both personal experience, and stories of others’ experiences:

*“I have heard awful stories of it being painful”. *Age 55–59/White/Southeast England.

*“IT FLIPPING HURTS!!! It’s the most excruciating pain I have ever experienced in my life and if you get a nurse who is impatient and unsympathetic*,* as I did*,* it’s even worse. Surely in this day and age there has to be a better way”.* Age 60–64/White/Yorkshire and the Humber.

Where free text comments contained information that could not be categorised into the pre-defined barrier items, additional codes were developed using inductive coding. Three of these additional barriers were mentioned by > 5% of the sample (Table 2). Work commitments that made it difficult to attend appointments were cited by 91/1074 (8.5%) of the respondents.

*“I am extremely busy at work and cannot take time off”. *Age 50–54/Asian/London.

Difficulties around appointment communication were described by 78/1074 (7.3%) of the respondents and included issues around rescheduling appointments or being able to get through to the breast screening service on the telephone.

*“Continuously routed to an answer machine to call back at an alternative time to rearrange invite. GP Surgery could also not assist with making my appointment. Unaccessible [sic] points of contact”. *Age 50-59/White/Southeast England.

Having a previous negative screening experience that was not related to pain or embarrassment was described by 55/1074 (5.5%) of the respondents.

*“I found the whole experience very uncomfortable the nurses were not empathic and rough while handling my breasts I would have thought and hoped that they would be more understanding and thoughtful. I know it probably isn’t the nicest of jobs to have to do seeing breasts all day however as a patient it isn’t a nice experience for us either”. *Age 60–64/Mixed Black Caribbean and White/Yorkshire and the Humber.

Additional barriers identified in the free text (mentioned by < 5% of the sample) are described in Additional File 5.

### Variation in barrier endorsement by demographic characteristics

Endorsement of each of the top six barriers by demographic characteristic is given in Table 3. There were several age differences in barrier endorsement. Women in the middle age groups (age 55–59 and 60–64) were more likely to endorse not wanting the test to be done by a man (age 55–59: 31.5%, age 60–64: 33.7% endorsement) compared to older (age 65–69: 20.9%, age 70+: 14.6%) and younger women (age ≤ 54: 24.8%) (χ^2^ (4, *N* = 1067) = 15.04, *p* < 0.01). Women aged 55–70 (age 55–59: 28.0%, age 60–64: 32.6%, age 65–69: 33.1%, age 70+: 26.8%) were more likely to endorse previous pain during screening as a barrier than women aged 54 or under (13.9%; χ^2^ (4, *N* = 1067) = 33.79, *p* < 0.001). Women aged ≤ 64 were more likely to endorse difficulty getting a convenient appointment than those aged over 64 (age < 55: 37.4%, age 55–59: 29.0%, age 60–64: 27.6% v age 65–69: 23.0%, age 70+: 22.0%); χ^2^ (4, *N* = 1067) = 14.01, *p* < 0.01).

**Table 3:** Endorsement of the top six barriers by demographic factor (% (*n*))

	Barrier endorsement by demographic factor (% (*n*))					
	Barrier 1^a^ Appt difficulty	Barrier 2 Concern man	Barrier 3 Worry about pain	Barrier 4 Previous exp of pain	Barrier 5 More important worries	Barrier 6 Appt too far
*Age groups (N* = 1067)^b^						
≤54 (*N* = 303)	37.6 (114)^*^	**24.8 (75)**	29.0 (88)	**13.9 (42)**	24.8 (75)	23.2 (70)
55–59 (*N* = 314)	**29.0 (91)**	**31.5 (99)**	31.5 (99)	**28.0 (88)**	22.0 (69)	23.2 (73)
60–64 (*N* = 261)	**27.6 (72)**	**33.7 (88)**	24.1 (63)	**32.6 (85)**	24.1 (63)	23.0 (60)
65–69 (*N* = 148)	**23.0(34)**	**20.9 (31)**	20.9 (31)	**33.1 (49)**	24.3 (36)	19.6 (29)
70+ (*N* = 41)	**22.0(9)**	**14.6 (6)**	24.4 (10)	**26.8(11)**	26.8(11)	26.8 (11)
*Ethnic group (N = 1045)*						
White (*N* = 973)	29.7 (289)	**27.1 (264)**	27.0 (263)	26.2 (255)	23.2 (226)	22.2 (216)
Other group (*N* = 72)	31.9 (23)	**40.3 (29)**	25.4 (19)	23.6 (17)	29.2 (21)	25.0 (18)
*Education (N = 930)*						
Degree or above (*N* = 341)	31.7 (108)	27.0 (92)	30.2 (103)	25.8 (88)	22.9 (78)	21.7 (74)
Below degree (*N* = 589)	27.8 (164)	27.7 (163)	26.3 (155)	26.0 (153)	24.4 (144)	22.9 (135)
*Self-reported disability? (N = 1006)*						
Yes (*N* = 282)	24.3 (73)	30.5 (86)	26.6 (75)	28.9 (73)	23.0 (65)	**28.4 (80)**
No (*N* = 274)	31.4 (227)	25.6 (185)	26.5 (192)	24.9 (180)	23.2 (168)	**19.3 (140)**
*Mental health condition? (N = 1017)*						
Yes (*N* = 242)	24.8 (60)	**33.5 (81)**	25.6 (62)	24.0 (58)	27.3 (66)	25.6 (62)
No (*N* = 775)	31.4 (243)	**25.9 (201)**	27.4 (212)	25.7 (199)	22.1 (171)	21.3 (165)

^a^Barrier 1 = difficulty getting an appointment. Barrier 2 = concern about a man doing the screening test. Barrier 3 = worry that breast screening will be painful. Barrier 4 = previous experience of pain during breast screening. Barrier 5 = having more important things to worry about. Barrier 6 = appointment too far from home.

^b^Prefer not to say and I don’t know responses were coded as missing and removed from the analysis so sample sizes for each analysis vary.

*Bold indicates significant different at *p*<.05.

Women from minoritised ethnic groups were more likely to endorse concerns about a man doing the screening test compared with women of white backgrounds (40.3% vs. 27.1% respectively; χ^2^ (1, *N* = 1045) = 5.74, *p* < 0.05).

Women reporting a mental health condition were more likely to endorse concern that a man may do the screening test than women who did not report a mental health condition (33.5% vs. 25.9% respectively; χ^2^ (1, *N* = 1017) = 5.23, *p* < 0.05). Women reporting a disability were more likely to endorse that the appointment was too far from home compared to women without a disability (28.4% vs. 19.3% respectively; χ^2^ (1, *N* = 1006) = 9.69, *p* < 0.01). There was little variation between any of the most endorsed barriers and level of education.

## Discussion

The most commonly endorsed barriers amongst non-attending women responding to our survey were difficulties making a convenient appointment close enough to home, worry about and/or previous experience of pain, concern that a man would do the test and having too many other things to worry about. In particular, pain (worry about pain and/or previous experience of pain) was a markedly prominent barrier, endorsed by nearly 40% of participants. These barriers are similar to those identified in the recent Cancer Awareness Measure (CAM) survey [4] where appointment convenience and locality, pain, concerns about a man doing the screening test and having too many other things to worry about also featured within the most endorsed barriers among screening attenders as well as non-attenders. Endorsement of the barriers by non-attenders (*N* = 91) in the CAM survey ranged between 9 and 19% which was lower than for this study and may reflect the different sampling methods (direct contact with non-attenders vs. a population-based survey including only a small proportion of non-attenders).

Exploration of the free text responses provided additional insight into barriers faced by participants. The high volume of free text responses was unanticipated, and it was interesting that women used this space as a forum to express their concerns and experiences about screening in more detail. A substantial proportion elaborated on experiences or fears about pain. This corresponds with both the findings of this study and existing quantitative and qualitative research illustrating that pain represents a particularly powerful barrier to different types of cancer screening [16–18]. The free text responses also revealed additional barriers, the most common of which were work commitments, difficulties with appointment communication and previous negative experience of screening. These are closely aligned with the pre-defined barriers of appointment convenience, pain and embarrassment and highlight the salience of issues around accessibility and acceptability.

Most women reported multiple barriers to breast screening which often encompassed facets of individual belief (e.g. worry about screening being painful) and motivation to attend screening (e.g. beliefs about having other more important things to do) as well as wider organisational and opportunistic barriers s (e.g. not being able to find a local appointment). These findings highlight the multifaceted causation of screening non-attendance and are consistent with the COM-B (Capability Opportunity Motivation– Behaviour) framework of health behaviour [19] which conceptualises screening behaviour as the product of an individual’s capability, motivation and opportunity to attend screening. This suggests that multi-component interventions that tackle a variety of barriers spanning individual beliefs around capability and motivation as well as addressing service-level barriers facilitating the opportunity to attend may be most useful to increase participation.

Exploratory analysis indicated variations in barrier endorsement according to several demographic factors. Age was found to have a significant influence on barrier endorsement. Women aged ≤ 64 reported greater difficulties getting a convenient appointment which may reflect greater demands on their time particularly from work. Older women were more likely to report previous experience of pain as a barrier, consistent with their greater breast screening experience. Prior experience of pain during breast screening is a significant predictor of expectation of pain at future breast screening and is significantly associated with reduced reattendance [18].

Age was also associated with concern that a man may conduct the mammogram with women aged between 55 and 64 more likely to endorse this barrier than younger or older women. There is no clear explanation for this finding. It may be spurious due to multiple testing in exploratory analysis but merits further research. Women from minoritised ethnic backgrounds and those who reported mental health problems were also more likely to be concerned about a man doing the mammogram​. Mammography is the only health procedure conducted exclusively by women practitioners with employers allowed to restrict mammography roles to females only within current equality legislation. The NHS breast screening invitation states that “Female staff will take your mammograms” and the accompanying leaflet states that “Mammograms are carried out by women called mammographers” and uses she/her pronouns to reference the mammographer as well as using a picture of a female mammographer. However, the findings of our survey and other research suggest this message has not reached all screening invitees [20, 21]. Our finding that women from minoritised ethnic backgrounds were more likely to endorse concern about a man doing the screening test has also been identified in other research and may reflect cultural and religious differences in modesty, acceptability of nudity and perceptions of indecency [20].

In relation to mental health status, research has identified that women with mental health problems or mental illness are less likely to attend breast screening, have poorer cancer outcomes and may have less awareness and understanding of the specifics of the screening process, and may be more likely to avoid medical examinations in general [13, 22–24]. It is possible that reduced awareness and understanding may increase vulnerability to the misperception that a man may conduct the mammography. It is important to note that our self-reported measure did not gather specific information about the type or severity of mental health problems, so it is difficult to draw specific conclusions; however, the overall findings suggest that the misperception that a man may conduct the screening is more prevalent amongst women with mental health problems.

The appointment being too far away was more likely to be endorsed by those reporting a physical disability. This finding fits in with previous research illustrating that physical barriers to healthcare including distance to appointments and lack of transport are particularly salient for individuals with disability [25, 26].

It is noteworthy that we did not identify any variation in barrier endorsement according to educational level suggesting that the nature of barriers faced by individuals with different educational backgrounds may not be significantly different. In the context of the robust association between lower educational attainment and lower screening uptake [9], it is possible that this association may be driven more by the additive impact of multiple barriers and lack of resources to overcome those barriers facing those from lower educational backgrounds rather than from variation in the type of barriers faced.

Most respondents reported being aware of breast screening and receiving invitations, however, respondents from minoritised ethnic backgrounds were more likely to indicate lack of awareness of breast screening or report not ever receiving an invitation. This finding aligns with previous research that has identified lower levels of cervical screening awareness and engagement as barriers to uptake amongst minoritised ethnic populations [27]. It is particularly notable that this was the case even in a sample of women who had recently been invited for screening, according to NBSS.

There are several limitations to the survey results. As anticipated, the response rate was very low with only 5.1% of those identified as not attending breast screening entering the survey which limits generalisability of our findings. The low response rate was partially due to the inability to contact over a third of identified participants due to the lack of a recorded mobile phone number. This is an important limitation as the lack of valid mobile telephone numbers recorded in NBSS is not random. Those with no recorded number are likely to be women who have limited contact with their GP, are unregistered with a GP or have no mobile phone access. These women may experience different barriers to the women with recorded mobile telephone numbers and merit further research. The high proportion of women without a mobile number recorded also has implications for the planned shift to digital app-based invitations [28]. Similarly, it was not possible to determine the type of breast screening invitation (timed or open appointment) that participants had received, and it may be that barriers vary by invitation type.

The majority of respondents were aged < 64 which limits generalisability of our findings to older populations whose barriers may differ; however, as attendance has been found to increase with increasing age, this may be a less significant limitation [4]. Furthermore, due to the anonymous nature of the survey, we were not able to use information from screening records in our analysis and subsequently were not able to determine individual screening histories (for example, how many previous appointments had been missed or attended) nor previous screening results or breast cancer diagnoses. Similarly, we asked participants to report whether they had mental health conditions and/or disability using a binary response format, however, we acknowledge that these questions are simplistic and do not capture the different types of mental health conditions and disability that may differentially impact access to screening. More nuanced analysis exploring the barriers faced by individuals with different screening histories, mental health conditions and types of disabilities would provide more specific insight into the barriers encountered by these populations, however, our analysis aimed to provide an overview of the barriers faced by non-attenders as well as exploration of variation in barrier endorsement according to broad demographic variables as a first step to identifying targets for intervention.

Generalisability to all non-attenders is further limited by possible self-selection bias whereby those opting to complete the survey may differ from those who chose not to complete it. Similarly, the survey required participants to recall the barriers preventing them from attending screening and these recollections may be inaccurate and vulnerable to recall bias. We aimed to reduce this risk by only including non-attenders who had been invited for screening within the last six months. In addition, the study was carried out over a 6-month period which means we cannot rule out the possibility that we did not capture seasonal barriers affecting attendance at different times of the year. However, exploration of the free text responses revealed very little mention of seasonal issues (coughs/colds, seasonal busyness or holidays) suggesting that these issues were not significant barriers.

A significant proportion of respondents from minoritised ethnic backgrounds were routed out of the barriers part of the survey because, on entering the survey, they indicated they were either unaware of screening or reported not receiving an invitation. This, combined with the overall small numbers initiating the survey, means that relatively few women from minoritised ethnic backgrounds completed the barriers items, again limiting generalisability. The survey was administered in English only, which limited accessibility for non-English speaking participants. The survey did includ assessment of language and ease of understanding of the invitation which indicated only a small proportion of participants did not speak English and very few reported trouble understanding the invitation. However, respondents with limited English may have found it difficult to complete the survey or may have been routed around this question by indicating lack of screening awareness or invitation receipt at the start of the survey. Older women from minoritised ethnic backgrounds have also been found to have less access to mobile phones and the internet which would have further limited the accessibility of the survey [29]. In the context that screening adherence is typically lower in minoritised ethnic populations [8, 30] further research using more accessible methods is required to clarify the barriers that are most prevalent in these populations.

Despite the low response rate and limited ethnic diversity, the sample size was considerably larger than that included in the CAM survey and provides insight into the barriers faced by an under-researched population.

Our findings have three key implications for increasing breast screening uptake. Firstly, there is a need for appointment booking modernisation and flexibility as the most common barrier to attendance was difficulty getting a convenient appointment. The difficulties associated with competing priorities, locality of appointment and communication around the appointment were also highly endorsed or referred to within the free text responses. Ensuring flexible appointments in local areas that are easy to access and schedule as well as simple to cancel and reschedule would help to overcome this barrier. Secondly, there is a need to communicate even more clearly to women that men will not be conducting their breast screening to reduce this misperception. This could involve adding a direct statement clarifying the female only nature of mammography in the leaflet accompanying the breast screening invitation or may entail exploring different modes of communication from traditional written information, for example, greater use of emphasis and visual images to more clearly disseminate this message. This is a particularly important for equitable access as our findings suggest that this barrier disproportionately impeded women from minoritised ethnic backgrounds and those reporting mental health problems who have lower screening uptake and poorer breast cancer outcomes. It is timely to note here that current shortfalls in mammographer recruitment have led to recent calls to allow men to practice as mammographers; however, our findings suggest that this should be approached with caution and more research is required to determine ways in which this could be addressed sensitively, for example, the use of chaperones or offering choice of practitioner gender. Our findings correspond with the results of recent commissioned work exploring the potential impact of the introduction of male mammographers into the NHS breast screening programme [31].

Thirdly, our finding that both previous experience of pain and fear of pain were significantly endorsed barriers as well as the high volume of free text responses describing painful experiences or significant fear of pain, suggests that tackling pain during breast screening is important to increasing uptake. Pain has been identified as a consistent barrier to screening and has been found to reduce attendance [18, 32]. Little is currently known about ways to prevent or reduce pain during screening and there is a need to address this gap within the research to identify effective ways to make screening more comfortable for women [33].

## Conclusions

Barriers to breast screening relating to appointment convenience and locality, competing priorities, concern about a man conducting the screening test as well as both fear of pain and previous experience of pain were the most endorsed in our survey of non-attending women. Our survey has identified that modifiable barriers around accessibility as well as the acceptability and experience of breast screening are obstacles to women taking part. These findings provide important insights to inform the development of NHS plans to increase breast screening uptake.

## Supplementary Information

Additional File 1. List of services participating in the survey.

Additional File 2. Full version of survey.

Additional File 3. Table A1 Demographic Characteristics of eligible non-attending women.

Additional File 4. Table A2 Comparative full demographic characteristics of those starting the survey and those completing the survey.

Additional File 5. Table A3 All additional barrier codes with descriptions and examples identified within the free text responses.

## Data Availability

Data is provided within the manuscript or supplementary information files. The data are available from the authors upon reasonable request and subject to approval from the National Health Breast Screening Service.

## Abbreviations

NHSBSP: National Health Service Breast Screening Programme
NBSS: National Breast Screening Service
CAM: Cancer Awareness Measure
COM-B: Capability Opportunity Motivation-Behaviour model
