# Supplementary material for: Understanding barriers to breast screening: an online survey of non-attenders as part of a service evaluation in the breast screening programme in England
Source: BMC Public Health. 2025 Jul 19;25:2509. doi: 10.1186/s12889-025-23691-3 (PMC12275263; doi:10.1186/s12889-025-23691-3)
Supplement: Supplementary file 1 — Additional File 1. List of services participating in the survey. [file 12889_2025_23691_MOESM1_ESM.docx]

**Additional File 1**

**Study setting**

The study took place in 15 participating breast screening services in England, as listed below:

1. Bolton Breast Screening
2. Manchester
3. Newcastle
4. Pennine
5. Leeds/Wakefield
6. Liverpool
7. Warrington, Halton, St Helens & Knowsley
8. Wirral and Chester
9. Nottingham City
10. City, Sandwell and Walsall
11. West of London
12. Southampton & Salisbury
13. West Berkshire
14. Maidstone and South West Kent
15. West Devon and East Cornwall
