# Supplementary material for: Understanding barriers to breast screening: an online survey of non-attenders as part of a service evaluation in the breast screening programme in England
Source: BMC Public Health. 2025 Jul 19;25:2509. doi: 10.1186/s12889-025-23691-3 (PMC12275263; doi:10.1186/s12889-025-23691-3)
Supplement: Supplementary file 2 — Additional File 2. Full version of survey. [file 12889_2025_23691_MOESM2_ESM.docx]

**Additional File 2**

**Full survey**

**Survey introduction text**

Thank you for agreeing to take part in our survey. We want to know why some women do not attend breast screening. We would be grateful if you could answer the following questions to help improve our service. By completing the questions in this survey, you are agreeing to us using the information for this purpose. Your responses will be completely anonymous. This means we have no way of linking your answers with your personal details. We will not ask for your name or any other information to allow us to identify you. We won’t contact you again about this survey and won’t provide feedback on your responses.

| 1. In England, all women aged 50-71 are invited by letter to have x-rays of their breasts every 3 years. This is called breast screening (also known as mammography or mammogram). Have you heard of breast screening? | |
| --- | --- |
| Yes |  |
| No |  |
| I don’t know |  |

| 1. Have you ever been invited for breast screening? | |
| --- | --- |
| Yes |  |
| No |  |
| I don’t know |  |

| 1. Have you ever attended breast screening? | |
| --- | --- |
| Yes |  |
| No |  |
| I don’t know |  |

| 1. When you were last invited for breast screening, which of these describes you best?   Please select one option | |
| --- | --- |
| I don’t remember getting an invitation |  |
| I haven’t thought about whether to go yet |  |
| I have thought about it but I haven’t decided whether to go or not yet |  |
| I have decided not to go |  |
| I do intend to go |  |
| I booked an appointment but did not attend |  |
| I attended an appointment |  |

| 1. Why have you not attended yet? | |
| --- | --- |
| I have not got around to it yet |  |
| I had forgotten about it |  |
| There were other reasons I have not gone |  |

| 1. Is English your first language? | |
| --- | --- |
| Yes |  |
| No |  |
| I don’t know |  |

| 1. When you were last invited for breast screening, was the invitation letter and leaflet easy to read and understand? | |
| --- | --- |
| Yes |  |
| No |  |
| I can’t remember |  |

| 1. Did this put you off going for screening? | |
| --- | --- |
| Yes |  |
| No |  |
| I can’t remember |  |

| 9. When you were last invited for breast screening, did any of these things put you off taking part? | |
| --- | --- |
| I have found breast screening painful or uncomfortable when I have been before | Yes/No |
| I was worried that breast screening might be painful | Yes/No |

| 10. When you were last invited for breast screening, did any of these things put you off taking part? | |
| --- | --- |
| I found it difficult to get an appointment at a convenient time | Yes/No |
| The appointment was too far from home | Yes/No |
| I could not afford to cover the costs related to having an appointment (e.g., transport, childcare, reduced pay/earnings) | Yes/No |
| I had other more important things to worry about than breast screening | Yes/No |

| 11. When you were last invited for breast screening, did any of these things put you off taking part? | |
| --- | --- |
| I was too embarrassed to go to breast screening | Yes/No |
| I didn’t want a man to do the screening test | Yes/No |
| I was worried about having to take my clothes off or that too much skin would be showing | Yes/No |
| I was too frightened of what the test might find | Yes/No |

| 12. When you were last invited for breast screening, did any of these things put you off taking part? | |
| --- | --- |
| I was worried about catching COVID-19 if I went for screening | Yes/No |
| I had symptoms that might have been related to COVID-19 | Yes/No |

| 13. When you were last invited for breast screening, did any of these things put you off taking part? | |
| --- | --- |
| I had no symptoms of breast cancer | Yes/No |
| I don’t think I’m at risk of breast cancer | Yes/No |
| I don’t think breast screening works | Yes/No |
| I don’t trust the NHS | Yes/No |
| After thinking about breast screening, I decided that the harms of taking part outweigh the benefits | Yes/No |

| 14. When you were last invited for breast screening, did any of these things put you off taking part? | |
| --- | --- |
| I have a mental health condition or a learning disability that makes it difficult for me to go to breast screening | Yes/No |
| I have a physical health condition or disability that makes it difficult for me to go to breast screening | Yes/No |

| 15. Were there any other reasons that put you off going for screening?  *Please describe in your own words but do not include any information that would identify you or anybody else* |
| --- |
| *Free text* |

We would like to ask some questions about you so that we can understand more about the people completing our survey

| 16. What is your age? | |
| --- | --- |
| Under 50 |  |
| 50-54 |  |
| 55-59 |  |
| 60-64 |  |
| 65-69 |  |
| 70 + |  |
| Prefer not to say |  |

| 17. What is your ethnic group?  Choose one option that best describes your ethnic group or background | |
| --- | --- |
| **White** |  |
| English/Welsh/Scottish/Northern Irish/British |  |
| Irish |  |
| Gypsy or Irish Traveller |  |
| Any other White background |  |
|  |  |
| **Mixed/Multiple ethnic groups** |  |
| White and black Caribbean |  |
| White and black African |  |
| White and Asian |  |
| Any other Mixed/Multiple ethnic background |  |
|  |  |
| **Asian/Asian British** |  |
| Indian |  |
| Pakistani |  |
| Bangladeshi |  |
| Chinese |  |
| Any other Asian background |  |
|  |  |
| **Black/ African/Caribbean/Black British** |  |
| African |  |
| Caribbean |  |
| Any other Black/African/Caribbean background  Please describe |  |
|  |  |
| **Other ethnic group** |  |
| Arab |  |
| Any other ethnic group |  |
|  |  |
| **Prefer not to say** |  |

| 18. What is the highest educational qualification that you have completed?  Please select one option | |
| --- | --- |
| Apprenticeship |  |
| Degree or above (including HND or HNC, NQV level 4 and above, teaching or nursing degree) |  |
| NVQ or equivalent (including BTEC General/National, OND or ONC, City and Guilds Craft) |  |
| AS, A-Levels or equivalent |  |
| GCSE, O-Levels or equivalent |  |
| Other qualifications |  |
| No qualifications |  |
| Prefer not to say |  |

| 19. Do you consider yourself to have a disability? | |
| --- | --- |
| Yes |  |
| No |  |
| I don’t know |  |
| Prefer not to say |  |

| 20. Have you previously been diagnosed with a mental health condition? | |
| --- | --- |
| Yes |  |
| No |  |
| I don’t know |  |
| Prefer not to say |  |

| 21. Which region of England do you live in? | |
| --- | --- |
| North East England |  |
| North West England |  |
| Yorkshire and the Humber |  |
| East Midlands |  |
| West Midlands |  |
| East of England |  |
| London |  |
| South East England |  |
| South West England |  |
| I’m not sure which is my region |  |
| Prefer not to say |  |

| 22. Will you go to breast screening the next time you are invited? | |
| --- | --- |
| Yes, definitely |  |
| Yes, probably |  |
| No, probably not |  |
| No, definitely not |  |
| I don’t know |  |
| Prefer not to say |  |

| 23. Would you like to book a breast screening appointment now? | |
| --- | --- |
| Yes |  |
| No |  |

| **Close Screen**  Thank you for taking part in our survey.  For further information about breast screening, please visit  <https://www.nhs.uk/conditions/breast-screening-mammogram/>  For more information on how to book an appointment, please visit  <https://www.nhs.uk/conditions/breast-screening-mammogram/>  Your responses will be completely anonymous. This means we have no way of linking your answers with your personal details.  NHS England’s Privacy Notice (<https://www.england.nhs.uk/contact-us/privacy-notice/>) describes how we use personal data and explains how you can contact us and invoke your rights as a data subject. We will process your information in accordance with the requirements of the Data Protection Act 2018.   We are collecting / processing personal data about you for the following purpose(s):   - To identify barriers to breast screening   The information we will collect includes:   - Your demographic information - Your survey answers    The legal bases for this processing are:   - Where we process your personal data, we use Article 6(1)(e): exercise of official authority   We collect your personal data for this purpose from:   - Your answers to the survey questions   Your personal data is received and used by:   - NHS England - Queen Mary University   Your data will be stored for the following period:   - Your information will be stored in line with NHS England’s retention schedule |
| --- |
