# Supplementary material for: Understanding barriers to breast screening: an online survey of non-attenders as part of a service evaluation in the breast screening programme in England
Source: BMC Public Health. 2025 Jul 19;25:2509. doi: 10.1186/s12889-025-23691-3 (PMC12275263; doi:10.1186/s12889-025-23691-3)
Supplement: Supplementary file 3 — Additional File 3. Table A1 Demographic Characteristics of eligible non-attending women. [file 12889_2025_23691_MOESM3_ESM.docx]

**Additional File 3**

Table A1: Demographic Characteristics of eligible non-attending women (N = 27,729)

|  | **N** | **%** |
| --- | --- | --- |
| **Age** |  |  |
| Under 50 | 712 | 2.6 |
| 50-54 | 7,848 | 28.3 |
| 55-59 | 7,421 | 26.8 |
| 60-64 | 6,262 | 22.6 |
| 65-69 | 4,603 | 16.6 |
| 70+ | 867 | 3.1 |
| *Missing* | *16* |  |
|  | |  |
| **Index of multiple deprivation (IMD) quintile** | |  |
| 1 (most deprived) | 10,710 | 38.6 |
| 2 | 5,631 | 20.4 |
| 3 | 4,557 | 16.5 |
| 4 | 3,945 | 14.3 |
| 5 (least deprived) | 2,730 | 9.9 |
| *Missing* | *156* |  |
|  |  |  |
| **Round type** |  |  |
| Incident | 10,476 | 37.8 |
| Prevalent | 17,253 | 62.2 |
