# Supplementary material for: Understanding barriers to breast screening: an online survey of non-attenders as part of a service evaluation in the breast screening programme in England
Source: BMC Public Health. 2025 Jul 19;25:2509. doi: 10.1186/s12889-025-23691-3 (PMC12275263; doi:10.1186/s12889-025-23691-3)
Supplement: Supplementary file 4 — Additional File 4. Table A2 Comparative full demographic characteristics of those starting the survey and those completing the survey. [file 12889_2025_23691_MOESM4_ESM.docx]

**Additional File 4**

Table A2: Comparative full demographic characteristics of those starting the survey and those completing the full survey

| **Demographic characteristics** | **Started survey**  **(N, %**) N = 1,429 | | **Completed full survey**  **(N, %)** N = 1,074 | |
| --- | --- | --- | --- | --- |
| **Age** | | | | |
| Under 50 | 30 | 2.1 | 12 | 1.1 |
| 50-54 | 386 | 27.0 | 291 | 27.1 |
| 55-59 | 402 | 28.1 | 314 | 29.2 |
| 60-64 | 351 | 24.6 | 261 | 24.3 |
| 65-69 | 193 | 13.5 | 148 | 13.8 |
| 70 + | 59 | 4.1 | 41 | 3.8 |
| Missing | 8 | 0.6 | 7 | 0.7 |
| **Ethnicity** | | | | |
| White | 1248 | 87.3 | 973 | 90.6 |
| Other ethnic groups (all) | 147 | 13.7 | 72 | 6.7 |
| *Mixed/Multiple* | *21* | *1.5* | *15* | *1.4* |
| *Asian/Asian British* | *78* | *5.5* | *34* | *3.2* |
| *Black/ African/Caribbean/Black British* | *32* | *2.2* | *16* | *1.5* |
| *Other ethnic group* | *16* | *1.1* | *7* | *0.7* |
| Missing | 34 |  | 29 |  |
| **Language** | | | | |
| English as first language | - | - | 1013 | 94.3 |
| English not first language | - | - | 61 | 5.7 |
| **Highest educational qualification** | | | | |
| Degree or above | 433 | 30.3 | 341 | 31.8 |
| Education below degree (all) | 803 | 56.2 | 589 | 54.8 |
| *NVQ or equivalent* | *196* | *13.7* | *145* | *13.5* |
| *AS, A-Levels or equivalent* | *104* | *7.3* | *85* | *7.9* |
| *GCSE, O-Levels or equivalent* | *263* | *18.4* | *195* | *18.2* |
| *Apprenticeship* | *13* | *0.9* | *7* | *0.7* |
| *Other qualifications* | *115* | *8.0* | *76* | *7.1* |
| *No qualifications* | *112* | *7.8* | *81* | *7.5* |
| *Missing* | *193* | *13.5* | *144* | *13.4* |
| **Disability status** | | | | |
| Disability | 364 | 25.5 | 282 | 26.3 |
| No disability | 977 | 68.4 | 724 | 67.4 |
| Missing – I don’t know/Prefer not to say | 79 | 5.5 | 68 | 6.3 |
| **Mental health status** | | | | |
| Mental health condition | 313 | 21.9 | 242 | 22.5 |
| No mental health condition | 1037 | 72.6 | 775 | 72.2 |
| *Missing – I don’t know/Prefer not to say* | *79* | *5.5* | *57* | *5.3* |
| **Region** | | | | |
| North East England | 45 | 3.1 | 33 | 3.1 |
| North West England | 307 | 21.5 | 230 | 21.4 |
| Yorkshire and the Humber | 398 | 27.9 | 288 | 26.8 |
| East Midlands | 56 | 3.9 | 40 | 3.7 |
| West Midlands | 136 | 9.5 | 89 | 8.3 |
| East of England | 6 | 0.4 | 3 | 0.3 |
| London | 70 | 4.9 | 50 | 4.7 |
| South East England | 180 | 12.6 | 158 | 14.7 |
| South West England | 158 | 11.1 | 130 | 12.1 |
| *Missing – I’m not sure/Prefer not to say* | *34* | *5.1* | *53* | *4.9* |
