# Supplementary material for: Understanding barriers to breast screening: an online survey of non-attenders as part of a service evaluation in the breast screening programme in England
Source: BMC Public Health. 2025 Jul 19;25:2509. doi: 10.1186/s12889-025-23691-3 (PMC12275263; doi:10.1186/s12889-025-23691-3)
Supplement: Supplementary file 5 — Additional File 5. Table A3 All additional barrier codes with descriptions and examples identified within the free text responses. [file 12889_2025_23691_MOESM5_ESM.docx]

**Additional File 5**

Table A3: All additional barrier codes with descriptions and examples identified within the free text responses

| **Barrier code** | **Barrier descriptions** | **Example** |
| --- | --- | --- |
| Alternative screening type | Would prefer an alternative type of screening (heat/ultrasound etc) | *“I believe that ultrasound screening is a far better procedure and less possible side effects”.* |
| Appointment booked | Appointment booked or mentions that they are going to book an appointment | *“I phoned and arranged to make an appointment in June after [an operation].”* |
| Appointment cancelled | appointment cancelled by service | *“My appointment was cancelled as the machine was broken”.* |
| Appointment communication | Difficulty with communicating around appointment including can't get through on the telephone, being unable to cancel or reschedule an appointment and any other problem with communications. | *“Was unable to get through on the phone”.* |
| Appointment ill | Had an acute illness or injury when appointment was due or family member ill or injured when appointment due | *“I could not attend as I was having treatment for kidney stones”.* |
| Appointment location issues | Location issues including inaccessible by public transport, embarrassed at attending in supermarket, huge hospital, no disabled facilities, can't get there (not parking or distance to appointment issues) | *“A bit embarrassed about attending a mobile unit in a supermarket carpark.”* |
| Away | Away when invite arrived or when appointment was scheduled for | *“I was away on holiday when my appointment letter arrived so missed the appointment”.* |
| Body issues | gender dysmorphia, body dysmorphia, previous abuse, dislikes breasts being touched, embarrassed by weight | *“I was sexually and physically abused as a child and have struggled to attend appointments that require undressing, even though I know the benefits.”* |
| Breast issues | Concerns relating to implants, piercings, scar tissue, breasts too small to scan | *“I have breast implants, and my boobs are VERY tender, to the point of painful, to touch, so the thought of them being squashed and flattened in the machine, absolutely terrifies me”* |
| Caring responsibilities | Caring responsibilities made attendance difficult | *“I find it hard as I care for my [relative] who is ill”* |
| Current cancer | Current cancer diagnosis | *“I have just been diagnosed with [other] cancer....  Getting treatment for this is my priority.  Once this has been successfully treated I intend to attend the breast screening”.* |
| Fatalism | Don't want to know, what will be will be attitude | *“I don't care if I get cancer. Rather not know”* |
| Forgot | Just forgot or did not get around to it | *“I forgot to make an appointment”* |
| General fear | Fear or anxiety about hospitals, medical issues, health | *“The reason is that I need to pluck up the courage , I am crippled with anxiety at this test”* |
| Prior positive screen | Recalled for investigation (current or past), previous false positive | *“They recalled me for more of an in-depth scan. I was only a short wait.. maybe 1 to 2 weeks. In that time I’d convinced myself that I had cancer, made a will and sorted out stuff for my children! Drastic I know! I worried myself sick. Turns out it’s just ‘one of those things’ that tissue can overlap and makes it difficult to interpret. Obviously I’m pleased everything was ok… I just don’t want to go through that again. I’ll attend if I have symptoms.”* |
| Invitation error | Believed they were invited in error | *“I already attended a breast screening a year ago. I was invited again recently so I thought that as breast screening took place every 3 years, the latest invitation was sent in error.”* |
| Language issues | Does not speak English |  |
| Lost invitation | Lost invitation, can’t recall an invitation, moved house | *“I can’t find my letter of invitation now and keep forgetting to find out how to get an appt.”* |
| My choice | Just do not want screening, don't like the thought of it, can't cope with it | *“Don't want to go. What you don't know etc. Personal choice.”* |
| Negative prior experience | Negative prior experience in a screening appointment (not primarily about pain or embarrassment) | *“Previous bad experience at screening, staff not understanding and abrupt”* |
| No treatment | Would not have treatment so no point in being screened | *“My [relative] died of cancer last year. [Relative] was having blood transfusions and chemotherapy for [time period]. Sitting next to [relative] all those times, I couldn’t do it if I was diagnosed with breast cancer.”* |
| Overdiagnosis | Any discussion of worry about overdiagnosis or false positive results | *“After reading info. It states that 75% of those given treatment are found not to need it. I am not prepared to put myself through that, therefore unless I suspect I have cancer don't see the point in wasting anyone's time or money on breast screening.”* |
| Parking issues | Parking issues including hard to find parking, no disabled parking | *“Parking is a joke”.* |
| Prior cancer | Diagnosed with cancer in the past | *“I already had breast cancer in the past (and I've got secondary cancer at the moment), so I didn't see the point in going to this”.* |
| Private screen | Attended a private screen | *“I am entitled to a full health screen with BUPA through my job, so I went for a mammogram via that route instead. I'm very pleased that I received a letter from the NHS to attend breast screening. I consider it very important”.* |
| Screened abroad | Screened in another country | *“I am living abroad at the moment and had my breast screening carried out in France.”* |
| Screened for another reasons | Screened for other reasons (e.g. cysts), had other screening e.g. PET/CT scan, has annual scan due to family history or high risk | *“I'm scanned regular as I'm going through melanoma cancer...head chest stomach n pelvis”.*  *“The main one was that I had just had an U/S and mammogram for a benign cyst and didn’t want to use resources unnecessarily.”* |
| Screening is harmful | Mammogram as potentially harmful (e.g. radiation), might cause cancer, squashing of breasts may be harmful | *“Mammography helps detect breast cancer, but in women with a genetic predisposition it may increase the risk of this disease, which is why I am afraid of it”.* |
| Work commitments | Work commitments that made it difficult to attend, feeling that they would be penalised for taking time off, being unable to take time off, too busy at work. | *“I work for myself and out of hours appointments would really help me as the screaming was also taking place a distance away.”* |
